# Adaptive wavefront correction structured illumination holographic tomography

**DOI:** 10.1038/s41598-019-46951-w

**Published:** 2019-07-19

**Authors:** Vinoth Balasubramani, Han-Yen Tu, Xin-Ji Lai, Chau-Jern Cheng

**Affiliations:** 1 Institute of Electro-Optical Science and Technology, National Taiwan Normal University, Taipei, 11677 Taiwan; 2 Department of Electrical Engineering, Chinese Culture University, Taipei, 11114 Taiwan

**Keywords:** Imaging and sensing, Adaptive optics, Interference microscopy

## Abstract

In this study, a novel adaptive wavefront correction (AWC) technique is implemented on a compactly developed structured illumination holographic tomography (SI-HT) system. We propose a mechanical movement-free compact scanning architecture for SI-HT systems with AWC, implemented by designing and displaying a series of computer-generated holograms (CGH) composed of blazed grating with phase Fresnel lens on a phase-only spatial light modulator (SLM). In the proposed SI-HT, the aberrations of the optical system are sensed by digital holography and are used to design the CGH-based AWC to compensate the phase aberrations of the tomographic imaging system. The proposed method was validated using a standard Siemens star target, its potential application was demonstrated using a live candida rugosa sample, and its label-free three-dimensional refractive index profile was generated at its subcellular level. The experimental results obtained reveal the ability of the proposed method to enhance the imaging performance in both lateral and axial directions.

## Introduction

Structured illumination microscopy (SIM) is a popular optical imaging technique used to achieve high resolution^1–6^, and recent studies have demonstrated its ability to achieve resolution-enhanced imaging of biological specimens^7–13^. In structured illumination (SI) techniques, SI patterns are generated through different methods using either coherent^10–20^ or incoherent sources^21^, and low-resolution moiré beat patterns are detected from the convolved signals of SI patterns with the high-frequency components of the sample. In general, SI uses a phase-shifting method to separate overlapped spatial frequencies using singular value decomposition (SVD) and a pseudoinverse approach^15,22^. The separated high spatial frequencies are corrected and synthesized, thus providing resolution-enhanced imaging^15,19^. In recent years, the SI method has been used as a substitute for the synthetic aperture (SA) method in digital holographic microscopy (DHM)^23^ to generate quantitative phase image and refractive index (RI) profiles of biological samples^24–29^. Furthermore, in DHM, wavefront aberrations induced from the illumination and detection process in the optical system can suppress the spatial resolution as well as the reconstructed image quality. Several numerical compensation and adaptive methods are available to correct aberrations during the post-image reconstruction process^30–34^. In DHM, a conventional adaptive optical element, such as a deformable mirror device (DM), is used for phase aberration correction, but the DM control relies on mechanical control with complex tuning procedures^35–37^. Another active element that may possibly be used for phase aberration correction is a spatial light modulator (SLM), which can also be used for aberration compensation^38,39^. Several theoretical studies have been conducted using conventional SI to study aberration formations and the implications of conventional SI systems, and these studies have proposed several correction methods based on DM or numerical-compensation methods^35–37,40,41^. To date, no other study has examined the implementation of compact mechanical movement-free holographic approaches for AWC in SI system; to our knowledge, this study is the first to develop such a SI holographic imaging system. The proposed AWC technique is implemented on a compactly developed mechanical movement-free scanning structured illumination holographic tomography (SI-HT) system. The SI-HT system subsequently developed has several advantages: First, compact mechanical movement-free SI scanning is achieved by designing and displaying the CGHs composed of blazed grating and a phase Fresnel lens onto the phase-only SLM. Second, a feedback process is integrated with digital holography to sense the phase aberrations of the tomographic imaging system; the same is used to compensate the phase aberrations using mechanical movement-free CGH-based AWC techniques to enhance tomographic imaging performance in lateral as well as axial directions. Furthermore, the phase error induced by the variation and misalignment of the imaging system or environment can be sensed and compensated using AWC techniques to promote the long-term stability during measurements. The proposed AWC-SI-HT system performance is evaluated using a standard Siemens star target. The prospective biological application is demonstrated using a live candida rugosa as a sample, and its label-free three-dimensional RI profile is generated at the subcellular level.

## Working Principle

**Figure 1:**
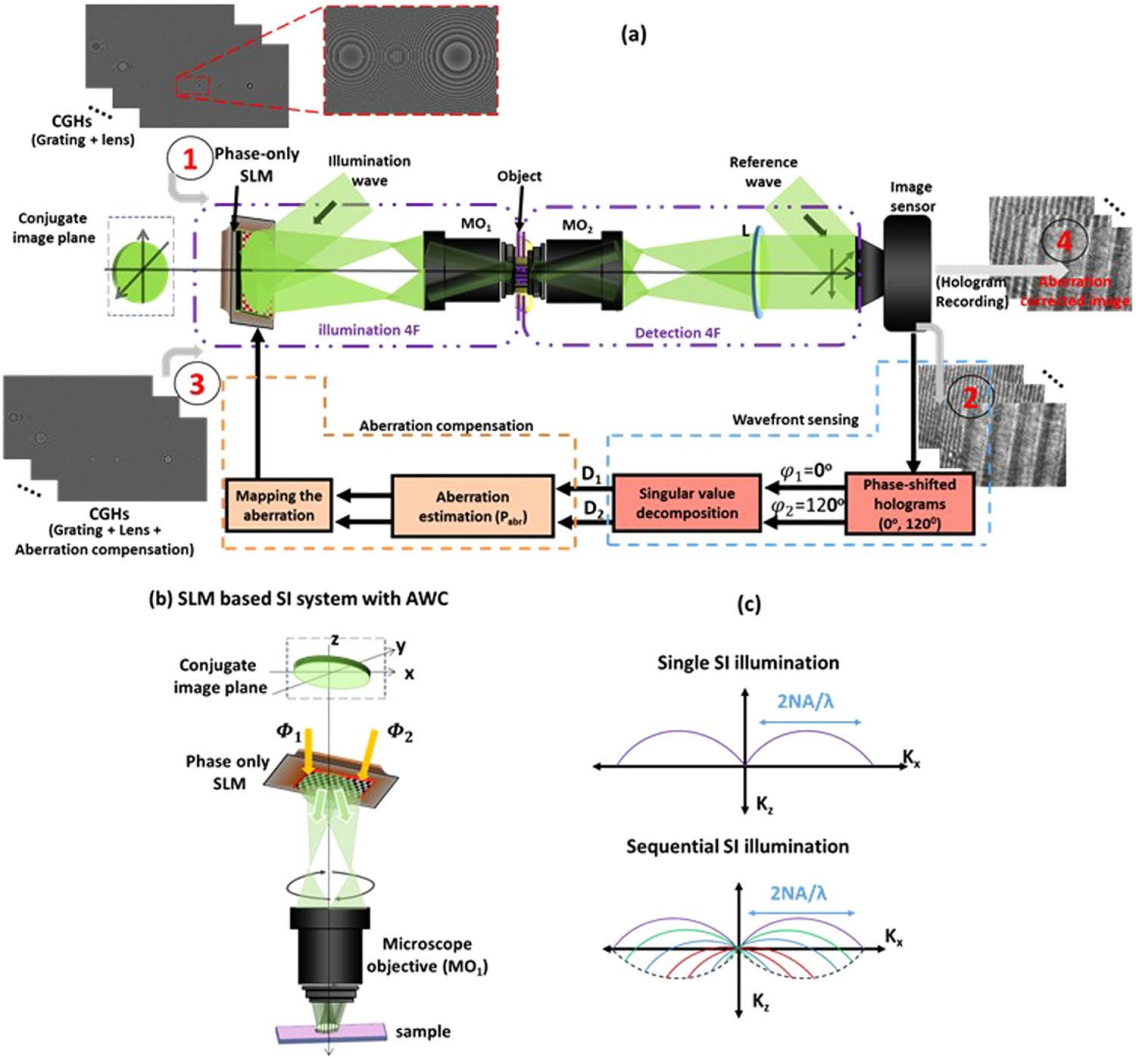
(**a**) Conceptual representation of AWC SI holographic tomography. (1) CGHs are displayed in phase-only SLM to execute SI scanning, and the corresponding SI holograms are recorded using an image sensor; (2) from the recorded holograms, the overlapped pass bands are separated, and the corresponding phase aberrations are estimated for all scanning angles; (3) new sequences of SI scanning CGHs are generated by mapping the collected aberration with a Zernike polynomial aberration model; and (4) holograms are recorded after aberration compensation and are used for further tomographic reconstruction. SLM (spatial light modulator), MO (microscope objective), and D_1_ and D_2_ are the passbands. (**b**) SLM based AWC SI-HT system, and (**c**) Spatial frequency coverages: single SI (upper); sequence of SI (lower).

The principle operation of the CGH-based AWC technique implemented on a compactly developed SI-HT system using a phase-only SLM is shown in Fig. 1(a). The operation of the system involves four major steps. First, CGH design for the compact scanning; second, wavefront aberration sensing; third, adaptive CGH design for AWC, and the final step involves the AWC calibrated SI-HT measurements and its image reconstructions.

In the first step, the key element to be considered for the CGH design is SLM. The main function of the phase-only SLM involves the development of the compact mechanical movement-free AWC SI-HT system. The conventional optical lens after the scanning element in the conventional SI system is replaced by the phase Fresnel lens as a CGH displayed on the SLM. The phase function of Fresnel lens can be expressed as, ${{\rm{\Phi }}}_{{\rm{FZL}}}(x,y)=\frac{\pi }{\lambda f}({x}^{2}+{y}^{2})$, where *λ* is the wavelength of the source, *x* and *y* are the spatial coordinates, and *f* is the focal length of the phase Fresnel lens. A compact mechanical movement-free scanning architecture are achieved by combining the binary blazed phase grating, ${{\Phi }}_{blazed}(x,y)=\frac{2\pi }{\wedge }(x+y)$ with phase Fresnel lens, and by rotating the grating period (Λ) the position of the probe beam are controlled in (*x*, *y*) directions^42^. The phase-only SLM are modulated by the phase functions Φ_1_and Φ_2_ to create SI as shown in Fig. 1(b). The expressions for *Φ*_1_and *Φ*_2_ are given as,

1 $${{\Phi }}_{1}(x)={{\Phi }}_{FZL}(x)+{{\Phi }}_{blazed}(x)$$

and

2 $${{\Phi }}_{2}(y)={{\Phi }}_{FZL}(y)+{{\Phi }}_{blazed}(y)$$

Then the developed CGHs are displayed on the phase-only SLM to achieve mechanical movement-free compact SI scanning, and the corresponding SI holograms are recorded using an image sensor. The frequency coverage corresponds to single SI and sequential SI imaging are shown in Fig. 1(d).

The second step involves the extraction of the phase aberrations from the recorded SI holograms. The wavefront distribution of the imaging system can be expressed as, $P(x,y)=\exp (j\frac{2\pi }{\lambda }{P}_{abr}(x,y))$, where P_*abr*_ is the phase aberration of the imaging system. The resultant wavefront (*R*_*SI*_ (*x*, *y*)) of the object excited with an SI pattern can be expressed as

3 $${R}_{SI}(x,y)=[{S}_{obj}(x,y)I(x,y)]\otimes P(x,y)$$

The corresponding SI spectrum in the frequency domain (${\tilde{R}}_{SI}(u,v)$) can be expressed as

4 $${\tilde{R}}_{SI}(u,v)=[{S}_{obj}(u,v)\otimes I(u,v)]CTF(u,v)$$

The symbols *S*_*obj*_ (*u*, *v*), *I* (*u*, *v*) and *CTF* (*u*, *v*) are the Fourier transforms of *S*_*obj*_ (*u*, *v*), *I* (*u*, *v*) and *P* (*x*, *y*), respectively. The illumination pattern is carefully controlled by *Φ*_1_ and *Φ*_2_. Accordingly, the SI wavefront spectrum (*O*_*SI*_ (*u*, *v*)) of the two overlapped passbands can be expressed as^18^.

5 $${O}_{SI}(u,v)={D}_{1}(u,v)+{D}_{2}(u,v)$$

The passbands corresponding to the SI illuminations are

6 $${D}_{1}(u,v)={A}_{i}(\,-\,{f}_{x},0){A}_{d}(u,v){S}_{obj}(u+{f}_{x},v)\,{P^{\prime} }_{abr}^{1}$$

and

7 $${D}_{2}(u,v)={A}_{i}(0,-\,{f}_{y}){A}_{d}(u,v){S}_{obj}(u,v+{f}_{y})\,{P^{\prime} }_{abr}^{2}$$

The symbols A_i_ and A_d_ are the transfer functions corresponding to the illumination and detection 4F systems of the object illumination^18^ as shown in Fig. 1(a), ${P^{\prime} }_{abr}^{1}\,$and $\,{P^{\prime} }_{abr}^{2}$ are the recorded aberrations, and the cut-off frequency, ${f}_{x,y}=\,\sin \,{\theta }_{x,y}/\lambda$, *θ*_*x*,*y*_ is the diffracted angle along *x* and *y* axes.

By subsequently solving the SVD and pseudoinverse approach the overlapped passbands are separated^15,19^. From the separated passbands, the phase aberrations, ${P}_{abr}^{1}(x,y)$ and ${P}_{abr}^{2}(x,y)$ are estimated iteratively for AWC. The third step in the operation of the system involves the CGH design for AWC. From the aberrations obtained, the corresponding compensation model is estimated at the conjugate image plane using a Zernike polynomial aberration model^38,43,44^, ${\tilde{P}}_{abr}={e}^{-j\frac{2\pi }{\lambda }(\sum _{p=0}^{q}{z}_{p})}$, where *p* is the polynomial order and *z*_*p*_ is the corresponding Zernike mode. Therefore, the estimated wavefront aberration *P*_*abr*_ (*x*, *y*) is numerically diffracted to the conjugate image plane and combined with Φ_1_ and Φ_2_ to develop CGH-based adaptive aberration-compensated imaging as shown in Fig. 1(a)^38^.

**Figure 2:**
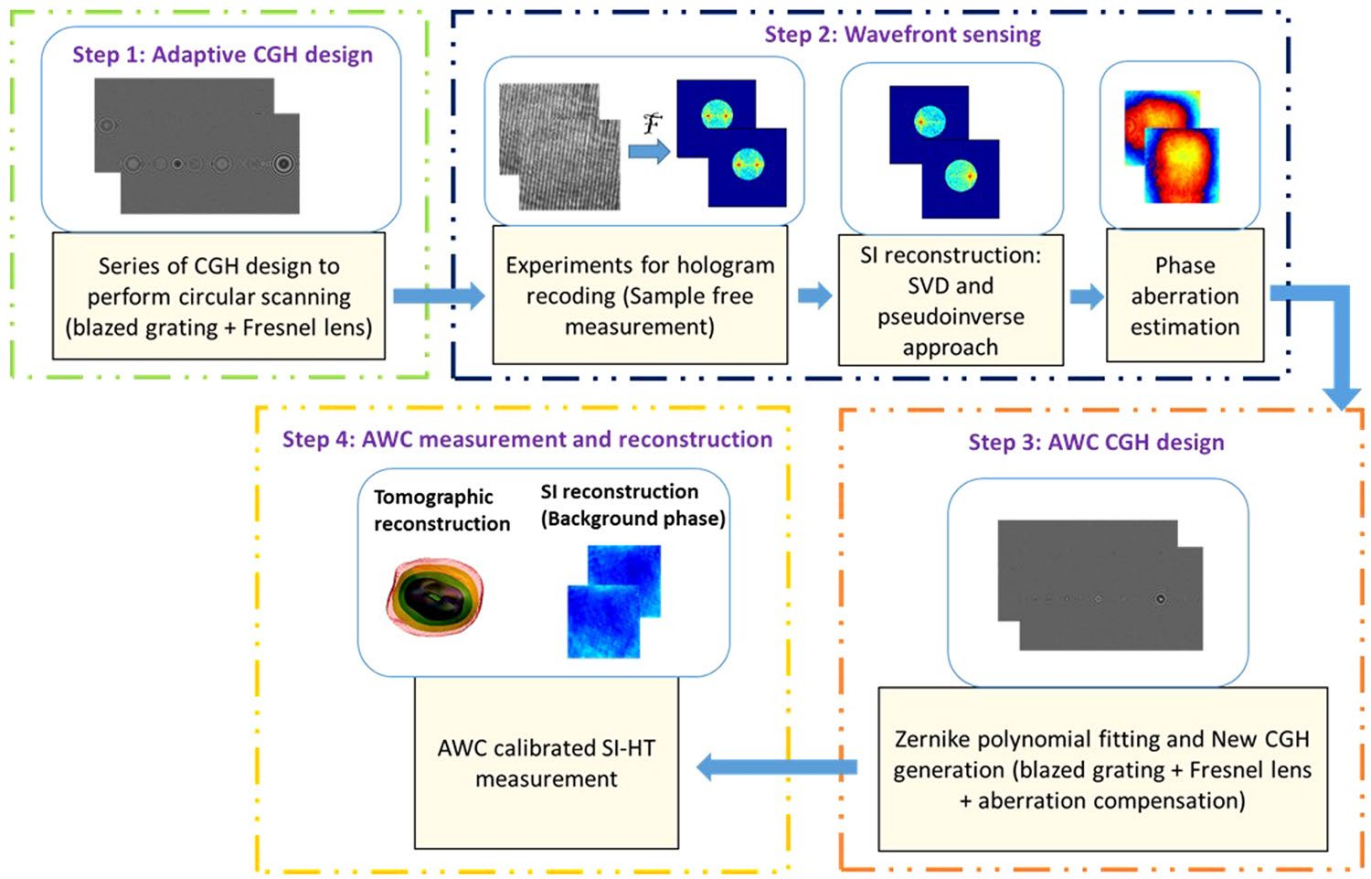
Calibrated measurement procedure of AWC SI-HT system listing each steps with experimental results.

Finally, step 4 consists of recording the SI holograms using the AWC CGHs designed in step 3. From the recorded holograms, as explained in the second step, the overlapped passbands are separated and synthesized to obtain an enlarged spatial frequency coverage. Thus, the resulting spatial frequency coverage (${R}_{o}(\zeta ,\eta )$) of the SI-HT system can be expressed as^18^

8 $${R}_{o}(\zeta ,\eta )=\sum _{m,n=1}^{M,N}[\{{A}_{i}(-{f}_{x}^{m},0){S}_{obj}(u+{f}_{x}^{m},v)\}+\{{A}_{i}(0,-\,{f}_{y}^{n}){S}_{obj}(u,v+{f}_{y}^{n})\}]\otimes {A}_{d}(u,v)$$

where, the symbol *m*, *n* denotes the number of passbands, and the symbol *M*, *N* denotes the number of hologram acquisitions. The calibrated measurement procedures of AWC SI-HT system showing each steps with its corresponding experimental results are described in Fig. 2.

## Experiments and Results

**Figure 3:**
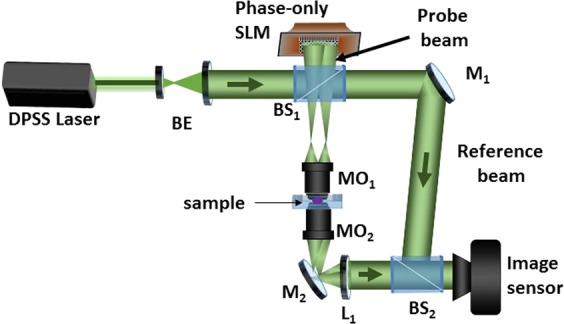
Experimental setup, BE: beam expander, BS: beam splitter, SLM: spatial light modulator, MO: solving the SVD matrix, the separated microscope objective, L: lens, M: mirror.

The CGH-based AWC SI-HT experimental setup follows a vertical type of modified off-axis Mach-Zehnder interferometric architecture. This configuration is more suitable for live sample measurement. A spatially filtered and collimated beam from a diode-pumped solid state laser (DPSS) emitting at 532 nm is used as a source and split by a beam splitter (BS) as a probe beam and a reference beam, as shown in Fig. 3. The phase-only SLM (Jasper Display corp., pixel number: 1920 × 1080, pixel size: 6.4 µm × 6.4 µm) is used to display the designed phase CGHs. The blazed grating profile is optimized in the CGH design and aligned the probe beam in order to avoid the zero order diffraction from the SLM.

**Figure 4:**
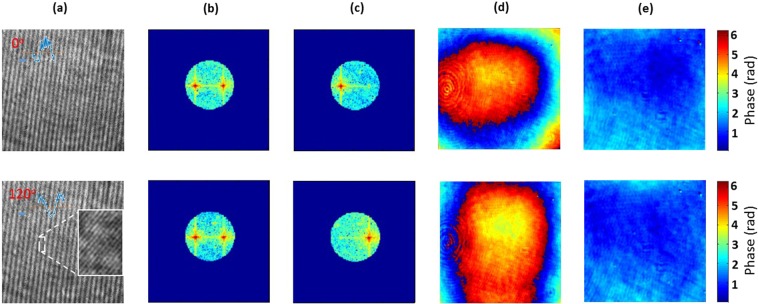
(**a**) Raw hologram corresponds to the phase shift of 0° and 120°, inset region shows Moire beat pattern. (**b**) First order frequency spectrum of (**a**) shows the overlapped frequencies. (**c**) Separated frequencies using singular value decomposition and pseudoinverse approach. Reconstructed phase: (**d**) before AWC and (**e**) after AWC.

**Figure 5:**
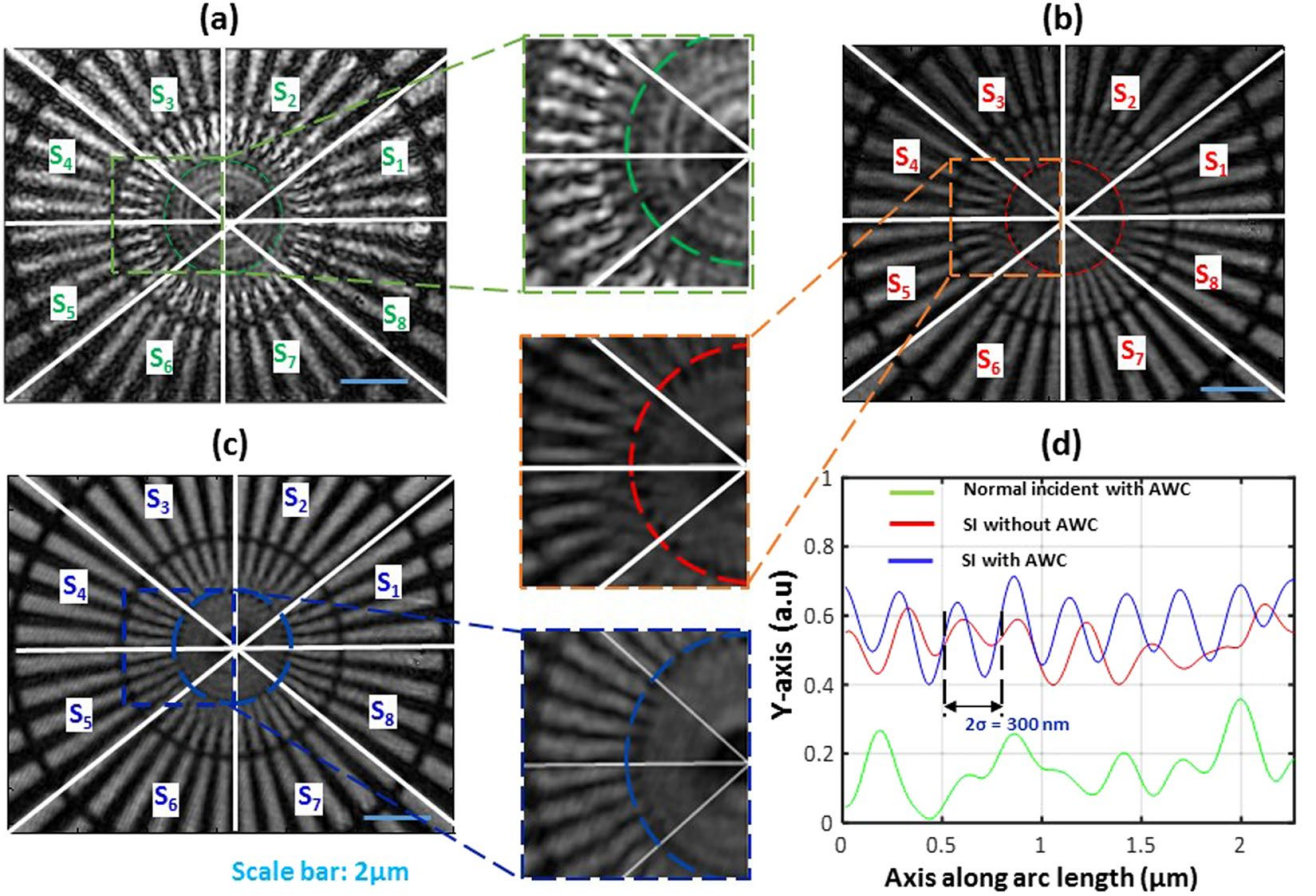
Siemens star target analysis. (**a**) normal incidence with AWC, (**b**) SI without AWC, (**c**) SI with AWC, (**d**) sectional profiles of ROI corresponds to (**a**–**c**).

The probe beam hits the phase-only SLM and reflects it back to create an SI pattern after the objective MO_1_ (NA = 0.9, 100x), and the beam passes through the sample. The wavefield with sample information is allowed to enter the detection 4F system (MO_2_: NA = 0.9, 100x, L1: 250 mm) and interfere with the reference beam. The background SI holograms with phase shifts corresponding to 0° and 120° are scanned along the circular directions, and the SI holograms are recorded using a complementary metal-oxide-semiconductor (CMOS) image sensor. The SI holograms and its frequency spectrums corresponding to the phase shifts of 0° and 120° are shown in Fig. 4(a,b), respectively. The separated passbands and corresponding phase aberrations are shown in Fig. 4(c,d) respectively. By then mapping back the estimated aberration using a Zernike polynomial model to iteratively generate a new CGH which is displayed on the SLM for AWC. The reconstructed phase profile after AWC are shown in Fig. 4(e), which shows that the proposed method can work well for the aberration correction. The quantitative amplitude validation of the proposed method is conducted using a standard Siemens star target as a test object which has a minimal line width of 150 nm (300 nm/pair). The experimental results are compared in Fig. 5. Different segments of target objects are marked from S_1_ to S_8_.

Figure 5(a) illustrates the normal aperture condition with an AWC scheme; for this case, the theoretical resolution at 532 nm can resolve only up to 455 nm, so high-frequency information cannot be resolved here. The SI scanning is performed in circular directions. The results without AWC and with AWC are compared in Fig. 5(b,c) respectively. Figure 5(b) shows that it can resolve only up to 230 nm, but our proposed AWC method can resolve high-frequency components of size 150 nm, as shown in Fig. 5(c). The cross-sectional profiles are compared in Fig. 5(d). The *σ* in Fig. 5(d) shows the resolvable resolution of the SI-HT system with AWC. We used 60 pairs of SI holograms (equivalent to 120 passbands) to achieve circular scanning within the numerical aperture of the objective lens. To achieve faster data acquisition, a LabVIEW-based software controller was developed to synchronize SLM and the image sensor, achieved a data acquisition time of less than 4 seconds.

**Figure 6:**
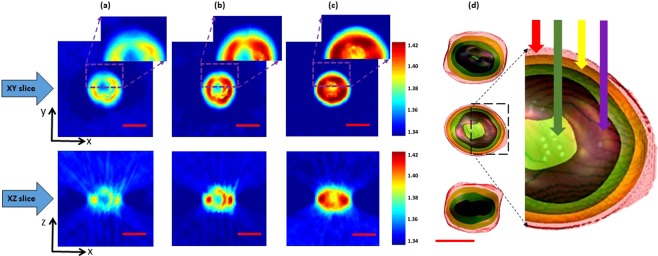
Reconstructed SI-HT slices, in *xy* and *xz* directions, of a live candida rugosa sample. Different comparisons are made between cases (**a**) with aberration, (**b**) with numerical aberration correction, and (**c**) with adaptive wavefront correction (AWC). The inset figures (**a**,**b**) illustrate the reconstructed cell quality for the comparisons, which shows that the AWC technique can achieve superior reconstruction performance; and (**d**) different 3D views correspond to 5(c), illustrating the subcellular structures of live candida rugosa. Arrow indications: red for the cell wall, green for mitochondria distribution, yellow for cytoplasm and purple for the nucleus. Scale bar: 2 µm. The color bar represents the quantitative RI values varies from 1.34 to 1.42.

For label-free RI tomographic image analysis, live candida rugosa (ATCC 14830) is used as a sample and is loaded into the microchannel (µ-Slide I^0.4^ Luer from ibidi) for the AWC SI-HT measurement procedures. The tomographic images are reconstructed^45,46^, and the different slices correspond to *xy* and *xz* directions, as elucidated in Fig. 6. Herein, the aberration data suffers to generate the cell structures; the cell wall and the inner organelles are not clearly visible, as illustrated in Fig. 6(a). Numerical aberration corrections were undertaken for the SI data, and the reconstructed results are shown in Fig. 6(b). It can resolve the cell wall and the inner structures but is still suffers to generate a high-quality sample profile compared with the proposed AWC technique shown in Fig. 6(c). After AWC was implemented, the cell structures and the cell’s inner organelles were observed clearly in *xy* and x*z* slices. The inset images are shown for all three cases in Fig. 6. This result demonstrates the ability of the proposed AWC to generate a high-quality sample profile in both lateral and axial directions and proves the potential applications for label-free imaging. Different 3D views are generated using the proposed AWC SI-HT approach. Based on the RI distribution the subcellular structures^28,45–49^, such as the cytoplasm, mitochondria distribution, and nuclei are clearly visible, as shown in Fig. 6(d). We posit that the proposed method could be used as a tool for generating a label-free 3D RI profile of a live sample.

## Conclusions

In summary, a novel CGH-based AWC technique was successfully demonstrated in the compactly developed mechanical-free scanning SI-HT system. The feasibility and the performance of the proposed method were studied in detail with a standard Siemens star target; the method was proven to resolve up to 150 nm, corresponding to a wavelength of 532 nm. Moreover, the method is not limited to the standard static targets but also demonstrated potential applications in label-free tomographic generation at subcellular levels using live candida rugosa samples (ATCC 14830). Therefore, it is expected that the proposed AWC SI-HT will be of use to the biomedical research community for the undertaking of further label-free quantitative analysis of native biological specimens at subcellular levels.
